# Abstract withdrawn by author

**DOI:** 10.1186/bcr3276

**Published:** 2012-11-09

## Abstract withdrawn by author

